# The Effects of Conservation Tillage on Chemical and Microbial Soil Parameters at Four Sites across Europe

**DOI:** 10.3390/plants11131747

**Published:** 2022-06-30

**Authors:** Ilka Engell, Deborah Linsler, Mignon Sandor, Rainer Georg Joergensen, Catharina Meinen, Martin Potthoff

**Affiliations:** 1 Centre of Biodiversity and Sustainable Land Use, Georg August-University Goettingen, Büsgenweg 1, 37077 Goettingen, Germany; linsler-d@arcor.de; 2 Department of Environmental and Plant Protection Engineering and Environmental Protection, University of Agricultural Sciences and Veterinary Medicine Cluj-Napoca, 3-5 Calea Manastur Street, 400372 Cluj-Napoca, Romania; sandor.mignon@usamvcluj.ro; 3 Soil Biology and Plant Nutrition, University of Kassel, Nordbahnhofstraße 1a, 7213 Witzenhausen, Germany; joerge@uni-kassel.de; 4 Department of Crop Sciences, Georg-August-University Goettingen, Von-Siebold-Straße 8, 37075 Goettingen, Germany; catharina.meinen@agr.uni-goettingen.de

**Keywords:** minimum tillage, no-tillage, mouldboard ploughing, soil quality, microbial biomass

## Abstract

Conservation tillage is often discussed as an effective tool to improve the soil quality in agriculture. Four sites across Europe (in Germany, Romania, Spain, and Sweden) were investigated as case studies for country-specific reductions in tillage intensity. Conventional tillage (CT) by mouldboard ploughing was compared with shallow and deep non-inversion minimum tillage (MT) and/or no-tillage (NT). In Sweden, NT and MT had positive effects on the concentrations of soil organic carbon (SOC), total nitrogen (N), and microbial biomass carbon (MBC) in the upper 20 cm compared with CT. At the German site, MT increased SOC, N, and MBC concentrations in the top 10 cm. In contrast, CT increased MBC contents and bulk density between 20 and 30 cm soil depth. At the Romanian site, soil parameters showed no differences between inverse tillage (CT) and non-inverse tillage (MT), both with a working depth of 25 to 30 cm. At the Spanish site, the use of NT significantly increased the concentrations as well as the stocks of C, N, and MBC compared to CT. In conclusion, reduced tillage improved soil microbial properties in most cases. However, the effectiveness of reduced tillage appears to be highly dependent on site conditions such as pH, soil texture, and climatic conditions.

## 1. Introduction

Conservation tillage has the potential to promote soil organisms [1], enhance the water infiltration capacity of soils, and reduce the risk of water erosion [2,3]. Further, the potential for carbon (C) sequestration is also a strong motivation for reducing tillage intensity in order to mitigate climate change [4,5]. Techniques of conservation tillage generally increase C and nitrogen (N) contents in the soil surface layer, thereby improving the soil structure and accelerating the soil’s resilience to extreme weather conditions [6]. In addition, tillage systems with a mulch layer reduce the risk of wind erosion [7,8] and lessen evaporation processes [9]. Nevertheless, conventional tillage (CT) is still a common tillage system in most European countries [10,11,12]. Main arguments against no-tillage (NT) systems are yield loss due to weed competition [13] and problems with seed germination [14].

The effectiveness of conservation tillage to improve soil conditions is still difficult to predict as there are significant differences among the various techniques grouped under this term. For instance, NT can mean direct seeding without any tillage operations [15,16] or opening a narrow trench for sowing [17]. Minimum tillage (MT) can be applied with various machinery (e.g., a disc harrow or a rotary harrow) at 5 cm [18], down to 8 cm [19], between 10 and 15 cm [20,21] or even down to 20 cm [22]. In contrast, CT is defined more uniformly in Europe, using a mouldboard plough at a working depth of between 20 and 30 cm [10,15,23].

In order to assess the advantages of conservation tillage over ploughing, indicators that reflect the status of soils are needed. Soil microbial properties as well as physicochemical soil characteristics are the two most important factors for the stability of a soil system [24]. To promote sustainable soil management and to improve soil fertility, an increase in surface-near nutrients and microbial biomass is recommended. The measurement of microbial biomass carbon (MBC) is a common tool to investigate the relationship between plant input, soil organic C (SOC) storage, nutrient mobilization and immobilisation processes [25,26]. MBC is often used in combination with measuring basal respiration, the mineralisation of SOC in the absence of fresh plant substrates [27,28]. In addition, the metabolic quotient *q*CO_2_ calculated as respiration-to-biomass ratio reflects the microbial demand for maintenance energy [29], whereas the ratio of MBC to SOC expresses the C availability for microorganisms [30]. Previous studies showed that conservation tillage, compared with ploughing, has the potential to increase MBC stocks and enhance microbial indicators [31,32,33,34]. In contrast, only moderate or even no increases in SOC stocks were found for non-inversion tillage treatments compared with mouldboard ploughing [21,35,36,37,38]. However, the effects seem to vary between sites and tillage regimes. 

Agricultural soils are influenced by the long-term history of cultivation practices and current management decisions of the farmer. Therefore, the present study follows a regional approach and investigates four sites, where tillage intensity was reduced in a country-specific way to match the demand of local management. The effects of conservation tillage systems on microbial, chemical, and physical soil parameters are compared with those of ploughing. The aim of this study was to find out which tillage system improves soil properties and thus enhances soil quality the most, taking into account site-specific conditions. The four sites form a climate gradient from Northern Europe (Sweden), via Central Europe (Germany) to South-Eastern Europe (Romania) and South-Western Europe (Spain), representing large areas of arable land in each country. The following four hypotheses were examined: (1) The positive effects of reducing tillage intensity on MBC are most visible in the upper soil and decrease with soil depth. (2) SOC stocks are not significantly different between conventional mouldboard ploughing and conservation tillage when regarding the whole sampled soil profile, whereas (3) MBC stocks are increased by a tillage reduction. (4) However, the effectiveness of conservation tillage regarding MBC dynamics depends on site-specific conditions such as soil texture, pH, and climatic conditions.

## 2. Results and Discussion

### 2.1. Säby, Sweden

**Figure 1:**
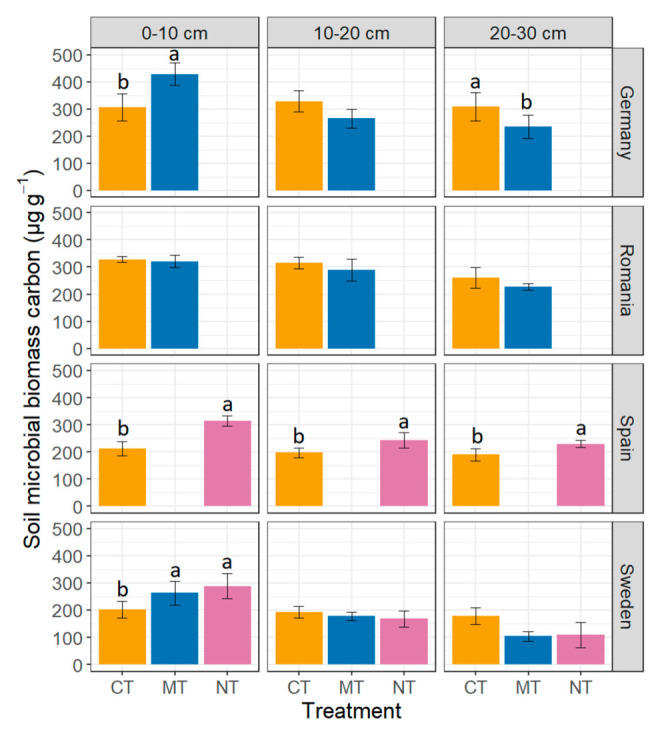
Effect of tillage treatments (CT = Conventional tillage, MT = Minimum tillage, NT = No-tillage) in Germany (*n* = 4), Romania (*n* = 3), Spain (*n* = 3) and Sweden (*n* = 3) on soil microbial biomass carbon at the soil depths (0–10 cm, 10–20 cm, 20–30 cm) given as means ± standard deviation. Means followed by different letters (a, b) are significantly (*p* < 0.05) different from each other at each soil depth. Tillage treatments were carried out in a site-specific way (Table 1).

In Sweden, SOC contents varied around 25.9 mg g^−1^ soil and declined by 34% from the top to the bottom layer under MT and by 50% under NT. In contrast, SOC concentrations at 0–20 cm under CT were nearly equally distributed with highest amounts in the deepest soil layer. At 20–30 cm, SOC contents were significantly affected by tillage treatments (F = 16.99; *p <* 0.05); contents of SOC were significantly higher (*p <* 0.05) at CT than at NT or MT plots (Table 1 and Table 2). Total N contents were in a range from 1.6 to 2.7 mg g^−1^ soil and were significantly influenced by tillage (F = 7.60; *p <* 0.05) at 10–20 cm. Soil samples at 10–20 cm soil depth from CT plots had greater total N contents (*p* = 0.05) than those from NT (Table 2). MBC contents were in a range from 61 to 333 µg g^−1^ and differed significantly (F = 52.77; *p <* 0.01) between tillage treatments at 0–10 cm soil depth; MT (*p <* 0.01) and NT (*p <* 0.01) enhanced MBC contents strongly compared with CT (Figure 1).

Summing up, the 11 years of MT and NT increased the contents of SOC, total N, and MBC in the top layers, accompanied by a strong depth decline in the bottom 20–30 cm layer. Similar depth declines have been repeatedly observed in Sweden [31,39]. The increase at 0–10 cm and the depth decline were most pronounced for MBC, indicating a closer relationship to the actual C input than SOC and N already stored in soil [1,40]. This effect was intensified by the strong bulk density (BD) increase from the 0–10 cm to the 10–20 cm layer. This increase occurred under all three tillage systems but especially under MT and NT (+40%) vs. CT (+30%). 

The BD varied around 1.02 g cm^−3^ at 0–10 cm and around 1.38 g cm^−3^ at 10–30 cm, without tillage effects at any depth (Table 2). The increased BD below followed from cultivation might form a barrier, which can reduce the C input by crop roots and, thus, MBC in the long-term [39,41,42]. The soil at this site, an acidic Eutric Cambisol, was characterised by high SOC stocks and low MBC stocks. Differences in SOC stocks were relatively strong between tillage treatments (F = 5.51; *p =* 0.07) whereas MBC stocks showed quite smaller variations (Table 3). The MBC/SOC ratio was around 0.7% among tillage treatments, which is most common in strongly acidic forest soils [29,43] and has rarely been measured in arable Cambisols [44], especially not in those with a relatively high clay content [25]. Acidification usually also increases the microbial demand for maintenance energy [29,45], which was reflected by high *q*CO_2_ values with a mean of 134 mg CO_2_-C g^−1^ MBC d^−1^ (0–30 cm soil depth). A high demand for maintenance energy lowers the MBC contents of a soil in the long-term [27,28]. However, low mean annual temperatures (MAT) and high mean annual precipitation (MAP) might also have reduced microbial decomposition of the annual C input, as indicated by the high SOC/total N ratio of 11.9. Overall, the study site at Säby is characterised by acidic conditions and low annual temperatures, high SOC contents and stocks. The low microbial availability of resources was reflected by low MBC/SOC ratios and quite small effects of tillage reduction on MBC contents.

**Table 1:** Overview of the different tillage treatments at the four field sites; CT = conventional tillage, MT = minimum tillage, NT = no-tillage.

Site	Tillage	Machinery Used (Working Depth)
Säby	CT	Mouldboard plough (23 cm)
	MT	Cultivator (10–12 cm)
	NT	Direct seeding without any tillage operations
Garte Süd	CT	Mouldboard plough (25–30 cm), followed by a rotary harrow
	MT	Rotary harrow (5–8 cm)
Turda	CT	Mouldboard plough (25–30 cm), seedbed preparation by a rotary harrow
	MT	Chisel processing (25–30 cm) after maize and wheat followed by disk harrow while direct seeding was applied after soybean
La Hampa	CT	Mouldboard plough (25–30 cm) plus cultivator (15–20 cm) and a disc harrow (15 cm)
	NT	Direct seeding without any tillage operations

**Table 2:** Mean (standard deviation) of bulk density, soil organic carbon (SOC) and total nitrogen (N) contents at the field sites Säby (*n* = 3), Garte Süd (*n* = 4), Turda (*n* = 3), and La Hampa (*n* = 3) under different tillage treatments (CT = conventional tillage, MT = minimum tillage, NT = no-tillage) at three soil depths (0–10 cm, 10–20 cm, 20–30 cm).

Site	Soil Depth	Bulk Density (g cm^−3^)			SOC (mg g^−1^ Soil)			Total N (mg g^−1^ Soil)		
	(cm)	CT	MT	NT	CT	MT	NT	CT	MT	NT
Säby	0–10	0.97 (0.10)	1.01 (0.14)	1.08 (0.16)	26.67 (1.51)	30.63 (1.55)	34.90 (4.19)	2.33 (0.05)	2.53 (0.09)	2.73 (0.19)
	10–20	1.25 (0.11)	1.41 (0.12)	1.50 (0.07)	27.00 (1.61)	26.13 (2.76)	23.53 (1.27)	2.30 (0.08) a	2.17 (0.12) ab	1.97 (0.05) b
	20–30	1.33 (0.18)	1.43 (0.07)	1.38 (0.13)	26.73 (2.40) a	20.17 (3.84) b	17.67 (3.73) b	2.20 (0.14)	1.77 (0.17)	1.63 (0.21)
Garte Süd	0–10	1.67 (0.12)	1.66 (0.10)		14.38 (1.48) b	18.28 (0.75) a		1.43 (0.04) b	1.75 (0.05) a	
	10–20	1.63 (0.05)	1.67 (0.09)		14.60 (0.99)	14.80 (0.94)		1.50 (0.00)	1.48 (0.04)	
	20–30	1.65 (0.05) b	1.83 (0.03) a		14.60 (2.42)	13.13 (1.61)		1.40 (0.07)	1.30 (0.00)	
Turda	0–10	0.89 (0.00)	0.85 (0.01)		22.13 (0.39)	20.67 (0.60)		2.13 (0.10)	2.00 (0.00)	
	10–20	0.98 (0.02)	0.88 (0.02)		22.53 (0.09)	20.87 (1.53)		2.13 (0.10)	2.00 (0.10)	
	20–30	1.01 (0.04)	0.90 (0.03)		22.43 (0.33)	19.53 (0.90)		2.13 (0.10)	1.90 (0.00)	
La Hampa	0–10	1.18 (0.29)		1.46 (0.19)	9.03 (0.48) b		10.30 (0.62) a	1.07 (0.05)		1.20 (0.08)
	10–20	1.29 (0.25)		1.26 (0.03)	8.23 (0.54)		9.13 (0.38)	0.90 (0.08)		1.07 (0.05)
	20–30	1.38 (0.05)		1.27 (0.16)	7.77 (0.05)		7.90 (0.65)	0.97 (0.05)		1.00 (0.08)

Different letters (a, b, ab) indicate a depth and site-specific significant difference between the tillage treatments (*p* < 0.05).

**Table 3:** Mean stocks (standard deviation) of soil organic carbon (SOC) and microbial biomass carbon (MBC) at the four field sites under different tillage treatments; CT = conventional tillage, MT = minimum tillage, NT = no-tillage. Information of tillage techniques are given in Table 1.

Site	Equivalent Soil Mass	SOC (t ha^−1^)			MBC (t ha^−1^)		
	(t ha^−1^ 0–30 cm)	CT	MT	NT	CT	MT	NT
Säby (*n* = 3)	3790	108.2 (1.5)	86.7 (7.4)	76.5 (13.7)	0.72 (0.09)	0.66 (0.04)	0.68 (0.12)
Garte Süd (*n* =4)	5060	75.3 (9.7)	76.2 (5.6)		1.62 (0.09)	1.53 (0.13)	
Turda (*n* = 3)	2760	59.0 (1.4)	58.8 (2.0)		0.83 (0.05)	0.77 (0.04)	
La Hampa (*n* = 3)	3790	32.5 (1.1) b		36.0 (1.1) a	0.78 (0.07) b		1.04 (0.02) a

Different letters (a, b) indicate a depth and site-specific significant difference between the tillage treatments (*p* < 0.05).

### 2.2. Garte Süd, Germany

Garte Süd is a long-lasting tillage experiment where the comparison between CT and MT already started 47 years ago [46]. Contents of SOC varied around 15.0 mg g^−1^ and the SOC/total N ratio was about 10 at Garte Süd. At 0–10 cm SOC contents were significantly (F = 36.07; *p <* 0.01) greater under MT compared with CT (Table 2). At the same soil depth, also total N contents were significantly greater (F = 169.00; *p <* 0.001) at MT plots than at CT plots (Table 1 and Table 2). MBC contents varied from 176 to 488 µg g^−1^ soil. In contrast to MT, the application of a mouldboard plough (CT) resulted in a homogenous distribution of SOC, total N and MBC concentrations in the sampled soil profile, presumably due to the strong mixing effect of this tool. At 0–10 cm soil depth, MBC contents were significantly (F = 14.50; *p <* 0.05) higher at MT compared with CT (Figure 1). Similar results have been repeatedly observed in Germany [32,37,41].

The soil at this site, a Haplic Luvisol, was characterised by high BD (mean of 1.69 g cm^−3^). At 20–30 cm soil depth BD was significantly (F = 31.23; *p <* 0.05) higher at MT compared with CT. The generally high BD is most likely caused by heavy machinery, especially for sugar beet harvesting in wet autumns [47]. Load-induced compaction is most likely the reason for the extremely high BD at 20–30 cm under MT. Generally, field crops have less difficulty with homogeneously high BD levels than with escalating increases [48]. This is in line with the study of Murugan et al. [32], who did not observe any yield difference between CT and MT at four sites in Germany for winter wheat and sugar beet.

This high BD led to the maximum equivalent soil mass, which is partly reflected by the SOC and MBC stocks. SOC and MBC stocks showed no differences between tillage practices (Table 3). In contrast, Heinze et al. [19] and Murugan et al. [32] observed approximately 10% higher SOC stocks and 20% higher MBC stocks on Luvisols in central Germany under MT in comparison with CT. However, these differences might be partly explained by a different sampling and calculation procedure [32]. The mean MBC/SOC ratio was 2.1% at Garte Süd, which is typical for central European Luvisols [19,32,37]. The mean metabolic quotient *q*CO_2_ value was on average 98 without significant differences between tillage practices. In conclusion, the field site Garte Süd is shaped by relatively high BD levels. The contents of SOC, total N and MBC differed in terms of tillage practices in the upper soil depth (0–10 cm), whereas stocks were not affected. 

### 2.3. Turda, Romania

Turda was the only site, where MT and CT were carried out on a similar working depth. The soil, a Phaeozem, was characterised by a high clay content (>50%) and a low BD level with a mean of 0.92 g cm^−3^. MBC contents (0–30 cm depth) varied around 290 µg g^−1^ soil (Figure 1). SOC contents were on average 21.4 mg g^−1^ soil at Turda with a mean SOC/total N ratio of 10 (Table 1 and Table 2). Among tillage treatments, total N contents ranged from 19 to 22 mg g^−1^ soil in the upper 30 cm. Phaeozems, typical for Romanian cropland, are known for their high natural fertility [49]. The clay content at Turda was considerably above that of other European Phaeozems [50,51], which led to generally low BD values and might improve its resilience against tillage-induced compaction [52,53]. 

The changes of MBC contents with depth were moderate for both tillage techniques (Figure 1). The MBC decline at 20–30 cm under CT indicates that the mouldboard plough did not always reach a working depth of 30 cm. In accordance with current results, no differences in SOC contents with depth between mouldboard ploughing and deep non-inversion chisel tillage have been measured on a Mollisol in Ohio, USA [54]. In contrast, much higher SOC contents have been observed in the top layers of a deep non-inversion tillage treatment than in a mouldboard ploughing treatment on a typical Ukrainian Chernozem [51]. This discrepancy cannot be explained by the current study. Other experiments did not find differences in SOC stocks between 12 and 25 cm deep non-inversion tillage [55]. For this reason, a reduced tillage depth should be tested at Turda, especially considering the low BD. The mean SOC and MBC stocks were relatively low at Turda, which points towards low C inputs at this site. This would lead to a starving and aged microbial community, as indicated by the relatively low *q*CO_2_ values [27,28]. This suggestion was confirmed by a mean metabolic quotient *q*CO_2_ of 59 at Turda. Also, the relatively low MBC/SOC ratios with a mean of 1.4% indicate a relatively low C availability to soil microorganisms [28,30]. In addition, strong bonding of relict SOC to clay minerals may further reduce C availability [56]. None of the soil physical (BD), chemical (SOC and total N) and microbial properties (MBC, MBC/SOC and *q*CO_2_) were strongly affected by tillage. This could be explained by the fact that both techniques were working at approximately the same depth (Table 1), which indicates that parameters depend more on working depth than on tillage techniques. The field site in Romania was characterised by high SOC concentrations, high clay contents and a low BD. The results from this site indicate that even different tillage techniques can lead to similar microbial conditions, which could by related to the soil type plus the choice of the same working depth for both machineries.

### 2.4. La Hampa, Spain

At the field site La Hampa, SOC contents were on average 8.7 mg g^−1^ soil with BD of around 1.31 g cm^−3^ (Table 1 and Table 2). SOC contents were significantly higher (F = 27.77; *p <* 0.05) in the upper 10 cm soil depth at NT plots compared with CT (Table 2). Total N contents (mean of 10 mg g^−1^ soil) were not affected by tillage reduction. MBC contents of NT plots exceeded those of CT plots (Figure 1) in the upper soil layer (F = 30.56; *p <* 0.05), at 10–20 cm (F = 33.04, *p <* 0.05) as well as in the lowest soil depth of 20–30 cm (F = 51.07; *p <* 0.05). The soil was characterised by high sand content and an alkaline soil pH and high MBC/SOC ratios (mean of 2.7%). Increased MBC/SOC ratios with increasing aridity of the climate have been repeatedly observed [57,58], due to shortening of the period for strong microbial activity. Consequently, *q*CO_2_ values were low at La Hampa with a range of 31.5 to 64.6 mg CO_2_ C g^−1^ MBC d^−1^, indicating a low demand of the microbial community for maintenance energy. This view is in line with the meta-analysis of Zuber and Villamil [59] who showed that sandy soils have lower *q*CO_2_ values under NT than under CT, whereas tillage effects were less in soils with finer particles.

The SOC stocks (mean of 32.5 t ha^−1^) were relatively low at this site. The latter was significantly greater under NT compared with CT for both, SOC (F = 339.97; *p <* 0.01) and MBC (F = 50.10; *p <* 0.05) (Table 3). These higher stocks were combined with a less pronounced depth gradient, which suggests a larger C input rate into the 10–30 cm layers at La Hampa, as proposed by Virto et al. [60]. Another reason for these positive NT effects on SOC and MBC stocks at this semi-arid region might be a slower turnover. Without mechanical disturbance under NT, the mineralisation of aggregate occluded SOC is most likely reduced [61,62]. This lowers the *q*CO_2_ values of a starving microbial population [32,63] followed by increased MBC contents and later by a higher contribution of microbial necromass-derived SOC.

Low *q*CO_2_ values also indicated that the low SOC stocks at La Hampa are not caused by microbial mineralisation but by low C inputs combined with a low microbial turnover. However, the possibility cannot be excluded that the SOC contents were already different at the start of the experiment, as sandy Fluvisols often exhibit a considerable sedimentation-induced spatial variability [44,57]. This is often not considered, as the initial soil properties are usually analysed by a so-called representative bulk sample, pooled from several cores and not from analysing each plot separately. Results from La Hampa in Spain, where the soil is characterised by a high sand content and an alkaline pH value, showed a strong positive effect of no-till management. This was reflected by higher MBC concentrations and stocks as well as greater SOC stocks. Low *q*CO_2_ values and a high MBC/SOC level indicate good conditions for microbial activity.

### 2.5. Effects among Sites

In general, the application of CT with a mouldboard plough resulted in a more homogeneous distribution of SOC, total N, and MBC contents, due to the strong mixing effect of this tool, whereas MT and especially NT created site-specific depth gradients. This decrease of approximately 30% from the top to the bottom soil layer was similar at Garte Süd and Säby. Hence, confirming our first hypothesis, most effects of reduced tillage on soil parameters were visible at the upper 10 cm soil depth, which was particularly strong at Garte Süd. Stocks of SOC and MBC were 34% higher and 55% lower, respectively, at Säby compared with Garte Süd. The difference in study duration might also explain differences between the site-specific effects. At the German site, reduced tillage was already applied for more than 40 years, whereas at the Swedish site it was just > 10 years. Based on 17 tillage experiments in the study from Smith et al. [64], SOC levels by sequestration needs 50 to 100 years to reach a new equilibrium. These differences are also reflected by the MBC/SOC ratio. Acidification is probably the main reason for the low MBC/SOC ratio at Säby [29], increasing the microbial demand for maintenance energy [45], which is also reflected by the high *q*CO_2_ values. In Romania deep non-inversion MT down to 25 cm had no specific positive or negative effects on soil parameters in comparison with CT, suggesting that shallow MT down to 10 cm should be used preferentially.

Contradicting our hypotheses two and three, SOC and MBC stocks were not affected in different ways by tillage. However, the effectiveness of conservation tillage on C stocks was lower than expected. Most evidently, La Hampa was the only site where the SOC and MBC stocks of the NT treatments significantly exceeded those of the CT treatment by 11% and 33%, respectively. SOC stocks at Säby were approximately three times higher, compared with Spain, but MBC stocks were slightly lower due to the acidic soil pH. The main reason for the positive NT effect at La Hampa is most likely the slower turnover of the microbial biomass due to drier climatic conditions, which was also reflected by low *q*CO_2_ values, indicating that the low SOC stocks at La Hampa are not the result of strong microbial mineralization. 

In line with our fourth hypothesis, the effectiveness of tillage reductions on soil parameters varied strongly between sites. Generally, less effects were found in Sweden, which is mostly related to soil pH and climate. As expected, the differences between the reduced tillage (MT/NT) systems were quite small at Säby. Results indicate that MT also has the potential to improve soil parameters to a similar extent as NT. In contrast, the strong effect of NT at La Hampa implies that no-till techniques are able to enhance microbial soil properties in semi-arid areas in a large extent. Further, marginal differences between tillage treatments in Romania (non-inversion vs. inversion tillage) indicate that soils with high clay contents and a good fertility might generally be affected less by tillage. 

## 3. Material and Methods

### 3.1. Field Sites Descriptions

Across Europe (Sweden, Germany, Romania, and Spain), four different long-term experimental field sites that focus on tillage were selected for sampling. As CT treatment, inversion mouldboard ploughing down to 30 cm was present in each country, whereas the reduced tillage treatments (MT and/or NT) varied in terms of machinery and working depths (Table 1). All sites are located on flat areas without inclination, so that they could not be affected by water erosion and colluvial processes.

In Sweden, the long-term experimental site Säby is located near Uppsala (59°49′ N 17°42′ E) and was established 11 years before sampling in 2006, using a randomized block design with a plot size of 9 × 20 m and three replicates. The mean annual temperature (MAT) at this site is 6.7 °C with 547 mm mean annual precipitation (MAP) (mean of the years 1988–2017). The soil is an Eutric Cambisol [65] with a soil texture of 25% sand, 52% silt and 23% clay [66] and a pH-H_2_O of 5.6. The crop rotation consisted of winter wheat (*Triticum aestivum* L.), oilseed rape (*Brassica napus* L.), and peas (*Pisum sativum* L.). Prior to sampling, winter wheat was sown in 2017 and 2016, and peas in 2015. Crop yields were 5.0 t ha^−1^, 5.6 t ha^−1^, and 4.2 t ha^−1^ in 2016, and 9.8 t ha^−1^, 9.9 t ha^−1^, and 9.0 t ha^−1^ in 2015 for CT, MT, and NT, respectively. The soil received mineral fertiliser depending on the cultivated crops, i.e., in total 139 kg N ha^−1^, 82 kg N ha^−1^, and 141 kg N ha^−1^ in the years 2017, 2016, and 2015, respectively.

In Germany, the long-term experimental site Garte Süd is located near Göttingen in southern Lower Saxony (51°29′ N 9°56′ E) and was established 47 years before sampling in 1970. The soil is a Haplic Luvisol [67] with 12% sand, 73% silt, and 15% clay [46] and a pH-H_2_O of 7.2. Average temperature is 9.5 °C MAT with a precipitation of 621 mm MAP (average from 1989–2018). Plots with a size of 20 × 40 m are arranged in a randomized block design with four replicates. Crop rotations varied inconsistently and were mainly based on cereals. In the two years before sampling, winter wheat (*Triticum aestivum* L.) in 2016 and a mixture of peas (*Pisum sativum* L.) and oat (*Avena sativa* L.) in 2015 were grown on the site. Crop yields were 7.7and 7.4 t ha^−1^ in 2016 as well as 3.5 and 3.0 t ha^−1^ in 2015 for CT and MT, respectively. As for fertilisation, Garte Süd received no inorganic fertiliser in 2015, 188 kg N ha^−1^ in spring 2016, and 207 kg N ha^−1^ in spring 2017.

In Romania, the long-term experimental site Turda is located near Cluj-Napoca (46°35′ N, 23°48′ E) as a combined tillage and crop rotation experiment with a plot size of 30 × 12 m and three replicates. It was established 11 years before sampling in 2007. The MAT is 9.0 °C at this site with 540 mm MAP [68]. The soil is a Phaeozem with 16% sand, 28% silt and 56% clay as soil texture [69] and a pH-H_2_O of 7.0. Crop rotations of both tillage systems were soy (*Glycine max* L.), winter wheat (*Triticum aestivum* L.), and maize (*Zea mays* L.). Crop yields were 7.3 and 7.4 t ha^−1^ in 2016 and 3.2 and 3.3 t ha^−1^ in 2015 for CT and MT, respectively. The crops were fertilised with 40 kg N ha^−1^ and 40 kg P ha^−1^ as complex fertiliser in autumn, while 30 kg N ha^−1^ was added as NH_4_NO_3_ in spring.

In Spain, the field site is part of the experimental farm La Hampa in the southwest near Sevilla (37°17′ N 6°3′ W). The trial was set up 10 years before sampling in 2008 as a randomized block design with a plot size of 14 × 22 m and three replicates per treatment. The average temperature is 19.0 °C MAT with 497 mm MAP [70]. The soil is a Calcic Fluvisol with a pH-H_2_O of 8.3. Soil texture is 58% sand, 18% silt and 24% clay [16]. The crop rotation contained cereals, sunflowers (*Helianthus annuus* L.) and legumes. The crops prior to sampling were winter durum wheat (*Triticum durum* L.) in 2017, broad bean (*Vicia faba* L.) in 2016 and again winter durum wheat in 2015. In 2016, crop yields were very low at 1.2 t ha^−1^ for CT and 0.1 t ha^−1^ for NT, due to extreme weather conditions, but in 2015 they were 4.6 t ha^−1^ for CT and 2.5 t ha^−1^ for NT. Wheat received a complex fertiliser at a rate of approximately 60 kg N ha^−1^, 26 kg P ha^−1^, and 50 kg K ha^−1^. Sunflowers and legumes were not fertilised.

### 3.2. Sampling and Soil Chemical Analysis

Samples were collected from 29 to 30 May 2017 at Garte Süd, from 12 to 22 June 2017 at Säby, from 15 to 25 May 2018 at Turda and on 04 April 2018 at La Hampa from three soil depths (0–10, 10–20, 20–30 cm). In all countries and years, samples were taken under flowering of winter wheat with a soil corer (5 cm diameter and 30 cm length). Four soil samples were taken from each plot and combined for analysis. SOC and total N were analysed from dried, sieved (<2 mm) and ball milled soil samples using a Vario Max CN elemental analyser (Elementar, Hanau, Germany). HCl was added to the soils from Turda and La Hampa to remove inorganic C, for the other field sites total C corresponds to SOC. Soil pH was detected in deionized water with a soil to solution ratio of 1:2.5. BD was calculated by dividing the soil core volume by the soil weight determined after drying the soil at 105 °C. 

### 3.3. Soil Biological Analysis

To determine MBC fumigation extraction [71] was used. Then, 10 g of field-moist soil were extracted pairwise, i.e., fumigated (24 h, ethanol-free CHCl3) and non-fumigated, with 40 mL of 0.05 K_2_SO_4_ [72]. Organic C in the extracts was measured using a multi N/C 2100S (Analytik Jena, Jena, Germany). MBC was calculated as *EC/k_EC_*, where *E_C_* = (organic C from fumigated soil sample)–(organic C from non-fumigated soil sample) and *k_EC_* = 0.45 [73]. Basal respiration was determined by the MicroResp method [74]. In brief, the soil was adjusted to a water content of 15% and stored at 22 °C for 3 days before measurements; soil equivalent to 400 mg dry soil was placed in 1.1 mL deep-well microtiter plates and incubated for 6 h in a closed system. The system includes a detection microtiter plate with a colorimetric CO_2_ trap containing 1% noble agar, 150 mM KCl, 2.5 mM NaHCO_3_ and 12.5 µg g^−1^ cresol red. The colour change in the CO_2_ trap was measured at the beginning (T0) and after 6 h of incubation (T6) at 570 nm with a microplate reader (BioTek, Winooski, USA). The difference in the absorption between T6 and T0 was converted into CO_2_-C (µg g^−1^ soil h^−1^). The metabolic quotient *q*CO_2_ was calculated as mg CO_2_-C g MBC^−1^ d^−1^.

### 3.4. Calculations and Statistical Analyses 

Data analyses were carried out using the statistical software R (version 3.6.1, R. Core Team, 2019). Stocks of SOC and MBC were calculated for equivalent soil masses to consider differences in BD [75]. All soil properties presented at 0–10, 10–20, and 20–30 cm as well as MBC and SOC stocks at 0–30 cm were analysed by linear mixed effect models using the package nlme (version 3.1-152, [76]). ‘Tillage’ was used as fixed factor, ‘block’ was considered as random factor. Analysis of variance was performed on the final models for each soil parameter. Residuals of the final model were checked for homoscedasticity. To examine significant differences between groups, a post-hoc test (Tukey test) was carried out, using the package lsmeans (version 2.30-0, [77]). All field sites were evaluated separately from each other. Results of the Romanian field site are presented in a descriptive way, because the experimental field site Turda was originally established as a combined crop and tillage experiment. Due to sampling only under one crop, randomization could not be secured as samples were only taken from winter wheat. Values in the text are given as mean ± standard deviation.

## 4. Conclusions

Comparing different practises of tillage reductions, our study showed that it is quite important to distinguish between different expressions of conservation tillage. Further, in order to give recommendations for tillage applications, the region, including climate and soil characteristics, has to be considered in each case. Therefore, direct comparison of different sites is limited, and environmental conditions must be considered when evaluating the effectiveness of conservation tillage systems. Especially in regions where agronomical disadvantages of NT could occur, other reduced tillage systems might be preferred without putting soil quality (as indicated by microbial properties) at risk. Therefore, the choice of machinery should also be based on other factors such as fuel consumption or harvest yields. However, our study showed that ploughless tillage systems are recommended as MT and NT resulted in an MBC, C, and N accumulation near the surface independent from site-specific conditions and appeared to have the potential to enhance microbial indicators as well as C stocks in most cases.

## Data Availability

Not applicable.
